# Association Between Elevated Neutrophil-to-Lymphocyte Ratio and Mortality Risk in Community-Acquired Pneumonia: A Systematic Review and Meta-Analysis

**DOI:** 10.7759/cureus.93292

**Published:** 2025-09-26

**Authors:** Zubair Ahmad Ganaie, Yousef Aqel, Bsher Almaalouli, Loiy Naser Alsarkhi, Albeena Kabir Dar, Abdelaziz Maali Abusal, Calvin R Wei, Adil Amin

**Affiliations:** 1 Internal Medicine, Medicity Hospital, New Delhi, IND; 2 Internal Medicine, Hamad Medical Corporation, Doha, QAT; 3 Faculty of Medicine, Damascus University, Damascus, SYR; 4 Internal Medicine, Kashmir Institute of Medical Sciences, Lawaypora, IND; 5 Internal Medicine, Hamad General Hospital, Doha, QAT; 6 Research and Development, Shing Huei Group, Taipei, TWN; 7 Cardiology, Pakistan Navy Station (PNS) Shifa, Karachi, PAK

**Keywords:** community-acquired pneumonia, meta-analysis, mortality, neutrophil-to-lymphocyte ratio, prognostic biomarker

## Abstract

The neutrophil-to-lymphocyte ratio (NLR), derived from routine complete blood count parameters, has emerged as a potential prognostic biomarker reflecting systemic inflammation and immune dysfunction. This systematic review and meta-analysis evaluated the association between elevated NLR and mortality risk in community-acquired pneumonia (CAP) patients. A comprehensive literature search was conducted across multiple databases from January 2011 to August 2025, identifying studies that measured NLR in adult CAP patients with mortality as an endpoint. Twelve observational studies were included, comprising diverse patient populations across different healthcare settings. Random-effects meta-analysis was performed using R software (R Foundation for Statistical Computing, Vienna, Austria), with relative risk as the primary effect measure. Quality assessment was conducted using the Newcastle-Ottawa Scale. The pooled analysis demonstrated that elevated NLR was significantly associated with increased all-cause mortality risk in CAP patients (RR: 2.02, 95% CI: 1.18-3.47, p<0.05). Patients with high NLR values had approximately twice the risk of death compared to those with lower ratios. Pooled analysis showed that high NLR was associated with increased risk of ICU admission (RR: 1.30, 95% CI: 1.11-1.53). Substantial heterogeneity was observed across studies (I² = 99% for mortality, 85% for ICU admission), likely reflecting variations in patient populations, NLR cutoff values, and clinical settings. Sensitivity analysis confirmed the robustness of findings. This meta-analysis supports NLR as a valuable, cost-effective prognostic biomarker for mortality prediction in CAP patients, potentially enhancing clinical decision-making when integrated with existing severity assessment tools.

## Introduction and background

Community-acquired pneumonia (CAP) remains a leading cause of hospitalization and mortality worldwide, with in-hospital mortality rates ranging from 5% to 17% in general populations and up to 32-49% in patients with severe CAP [1-2]. Despite advances in diagnostic techniques, antimicrobial therapy, and supportive care, accurate prognostication in CAP patients continues to be challenging, necessitating the identification of reliable, cost-effective biomarkers that can guide clinical decision-making and improve patient outcomes.

Traditional pneumonia severity scoring systems, including the Pneumonia Severity Index (PSI) and CURB-65 (Confusion, Respiratory rate, Blood pressure, 65 years of age and older), have been developed to assess disease severity and predict mortality risk [3-4]. However, these scoring systems demonstrate decreased discriminatory power with increasing age and may not adequately capture the complex inflammatory response underlying pneumonia pathogenesis [5-6]. Inflammatory biomarkers such as C-reactive protein (CRP) and procalcitonin have been investigated to enhance prognostic accuracy, but these markers are not always reliable and may be costly or unavailable in resource-limited settings [7-8].

The neutrophil-to-lymphocyte ratio (NLR), calculated as the simple ratio between absolute neutrophil count and absolute lymphocyte count from routine complete blood count, has emerged as a promising prognostic biomarker in various inflammatory conditions and infectious diseases [9-10]. NLR conjugates two faces of the immune system: the innate immune response, mainly due to neutrophils, and adaptive immunity represented by lymphocytes [11]. Under pathological stress, neutrophil counts typically increase as part of the acute inflammatory response, while lymphocyte counts simultaneously decrease due to stress-induced apoptosis and redistribution, resulting in an elevated NLR that reflects the severity of systemic inflammation [12-13].

In the context of pneumonia, neutrophils serve as key mediators of the innate immune response, rapidly mobilizing to infection sites to eliminate pathogens, while lymphocytes play crucial roles in adaptive immunity and long-term immune memory formation [14]. The balance between these cell populations, as reflected by the NLR, may provide valuable insights into disease severity, treatment response, and clinical outcomes in CAP patients.

Previous studies have demonstrated that NLR with a cutoff value greater than 10 was associated with higher mortality compared to traditional biomarkers including CRP, white blood cell count, neutrophil count, lymphocyte level, PSI, procalcitonin, and CURB-65 in patients with CAP [15]. However, individual studies have shown conflicting results regarding the independent prognostic value of NLR and its ability to enhance existing severity scoring systems. Some investigations suggest that NLR independently predicts adverse outcomes including mortality, intensive care unit admission, and prolonged hospitalization [16-17], while others report limited additional value when combined with established severity scores [18-19].

Given the heterogeneity in study populations, methodological approaches, and clinical outcomes across individual studies, a comprehensive meta-analysis is warranted to synthesize the available evidence and provide definitive conclusions regarding the association between NLR and mortality in CAP patients. This meta-analysis aims to evaluate the prognostic significance of NLR in predicting mortality among patients with community-acquired pneumonia and to determine whether NLR can serve as a reliable, easily obtainable biomarker to guide clinical management and improve patient outcomes.

## Review

Methodology

This systematic review and meta-analysis was conducted following the Preferred Reporting Items for Systematic Reviews and Meta-Analyses (PRISMA) 2020 guidelines.

Literature Search and Search Strategy

A comprehensive literature search was performed across multiple electronic databases from 1st January 2011 to 5th August 2025, including PubMed/MEDLINE, Embase, Web of Science, Cochrane Central Register of Controlled Trials (CENTRAL), and Scopus. The search strategy was developed using a combination of Medical Subject Headings (MeSH) terms and free-text keywords related to neutrophil-to-lymphocyte ratio, community-acquired pneumonia, and mortality. Primary search terms included population terms ("community-acquired pneumonia" OR "CAP" OR "pneumonia"), exposure terms ("neutrophil-to-lymphocyte ratio" OR "NLR" OR "neutrophil lymphocyte ratio"), and outcome terms ("mortality" OR "death" OR "survival" OR "prognosis" OR "outcome"). No language restrictions were applied, but studies were limited to human subjects. Additional search methods included manual review of reference lists from included studies and relevant review articles, forward citation searching of key studies using Google Scholar, conference abstracts.

Study Selection

Studies were included if they met the following criteria: observational studies (cohort, case-control, cross-sectional) and randomized controlled trials involving adult patients (≥18 years) diagnosed with community-acquired pneumonia, measurement of neutrophil-to-lymphocyte ratio at hospital admission or within 24 hours of presentation, all-cause mortality as primary or secondary endpoint, sufficient data to calculate relative risk, odds ratio, or hazard ratio with 95% confidence intervals.

Exclusion criteria included pediatric studies (patients <18 years), hospital-acquired pneumonia, ventilator-associated pneumonia, or healthcare-associated pneumonia, studies focusing exclusively on specific pneumonia etiologies (e.g., COVID-19 pneumonia) unless mixed populations included, case reports, case series with <10 patients, editorials, letters, and narrative reviews, studies with insufficient data for meta-analysis after attempting to contact authors, and duplicate publications or overlapping study populations. Two independent reviewers conducted the study selection process in two phases: title and abstract screening of all retrieved citations for potential relevance, followed by full-text evaluation of potentially eligible studies for final inclusion. Disagreements between reviewers were resolved through discussion, and when necessary, consultation with a third reviewer. The selection process was documented using a PRISMA flow diagram.

Data Extraction

Data extraction was performed independently by two reviewers using a standardized, pre-piloted data extraction form. Study characteristics extracted included first author and publication year, country, duration of follow-up, sample size, and patient demographics. Population characteristics included age, gender distribution, and comorbidities. Outcome measures extracted comprised the definition of mortality outcome, the number of events, and the total participants.

Quality Assessment

The methodological quality of included studies was assessed using the Newcastle-Ottawa Scale (NOS) for observational studies [20]. The NOS evaluates studies across three domains: selection of study groups (maximum 4 stars), comparability of groups (maximum 2 stars), and assessment of outcome (maximum 3 stars). Studies were classified as high quality (7-9 stars), moderate quality (5-6 stars), or low quality (<5 stars).

Data Analysis

All statistical analyses were performed using R software (R Foundation for Statistical Computing, Vienna, Austria), using the "meta" and "metafor" packages. The primary effect measure was relative risk (RR) with 95% confidence intervals. Random-effects meta-analysis was performed. A p-value less than 0.05 was considered significant. Statistical heterogeneity was assessed using Cochran's Q test (P-value <0.10 considered statistically significant), I² statistic (quantified as low [25-49%], moderate [50-74%], or high [≥75%] heterogeneity). Sensitivity analysis was performed by removing one study at a time and RR with 95% CI were reported.

Results

**Figure 1:**
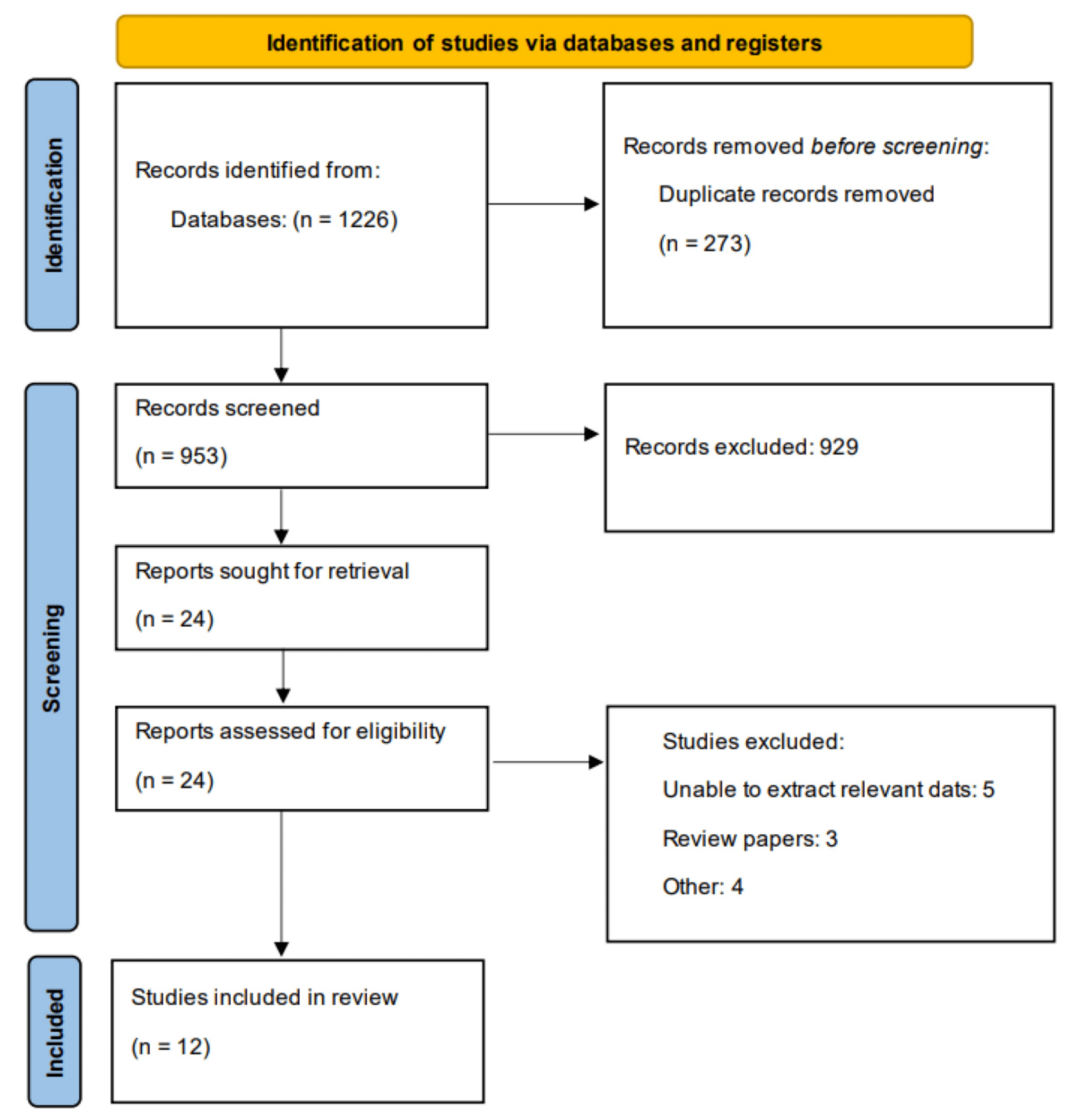
PRISMA flowchart of study selection

As a result of the literature search, a total of 1226 studies were identified. After removing 273 duplicates, the remaining studies were initially screened using their title and abstract. Full text of 24 studies were obtained, and a detailed assessment was done based on pre-defined inclusion and exclusion criteria. Finally, 12 studies were included in this meta-analysis. Figure 1 shows the PRISMA flowchart showing the detailed study selection process. Table 1 presents characteristics of included studies. Included studies published between 2012 to 2025. Table 2 presents the quality assessment of included studies.

**Table 1:** Included studies characteristics NR: Not reported; NS: Not specified; CKD: Chronic kidney disease; CVD: Cardiovascular disease; NLR: Neutrophil to lymphocyte ratio

Author	Year	Country	Cut-off	Sample Size	Follow-up	Death	Age	Male (n)	CKD (n)	Diabetes (n)	Hypertension (n)	CVD (n)
Cataudella et al. [21]	2014	Italy	NLR>=13.4	195	30 Days	49	NR	120	NR	60	110	NR
Cheng et al. [22]	2020	China	NLR>= 4	1360	In-hospital	129	67.21	850	170	1360	234	223
Cui et al. [23]	2024	China	NLR>=11.91	554	In-hospital	141	71	383	119	112	234	223
Curbelo et al. [24]	2017	Spain	NLR>10	154	30 Days	12	75.74	89	22	25	149	29
Feng et al. [25]	2021	China	NS	2028	In-hospital	121	76.93	1267	NR	486	NR	NR
Huang et al. [26]	2025	China	NLR>6.5	815	30 Days	56	76.36	509	112	253	490	179
Jager et al. [27]	2012	Netherland	NLR>=10	395	In-hospital	23	63.4	240	NR	68	NR	94
Lee et al. [28]	2021	Korea	NS	175	30 days	15	67.7	124	7	33	43	37
Ozmen et al. [29]	2016	Turkey	NS	143	In-hospital	27	73	83	NR	34	62	90
Sharma et al. [30]	2025	Australia	NLR >12	7862	In-hospital	321	618	4290	1379	NR	NR	NR
Tekin et al. [19]	2024	United States	NLR >12	4039	In-hospital	128	78	2172	1159	1220	NR	1118
Yang et al. [31]	2017	China	NLR>7.12	318	In-hospital	23	61	211	NR	48	73	21

**Table 2:** Quality assessment of included studies

Author	Selection	Comparison	Assessment	Overall
Cataudella et al. [21]	4	2	3	Good
Cheng et al. [22]	3	2	3	Good
Cui et al. [23]	3	1	3	Good
Curbelo et al. [24]	4	2	2	Good
Feng et al. [25]	4	2	3	Good
Huang et al. [26]	4	2	3	Good
Jager et al. [27]	4	1	3	Good
Lee et al. [28]	4	2	3	Good
Ozmen et al. [29]	4	2	3	Good
Sharma et al. [30]	3	2	3	Good
Tekin et al. [19]	4	1	3	Good
Yang et al. [31]	3	2	3	Good

Effect of NLR on Mortality

**Figure 2:**
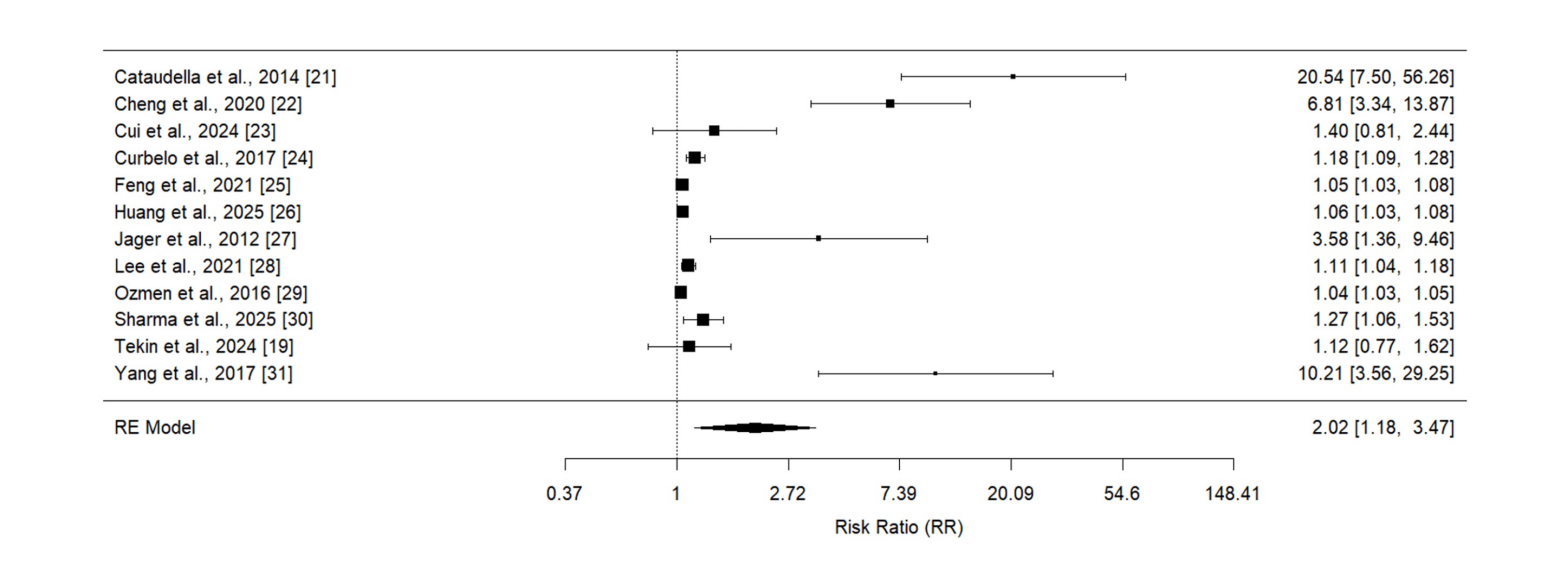
Effect of NLR on mortality References [19, 21-31]

Twelve studies were included in the pooled analysis showing the effect of high NLR on mortality in subjects with CAP, and the results of pooled analysis are shown in Figure 2. Pooled analysis showed that the risk of all-cause mortality is significantly higher in subjects with high NLR (RR: 2.02, 95% CI: 1.18 to 3.47). High heterogeneity was reported among the study results (I-Square: 99%).

**Figure 3:**
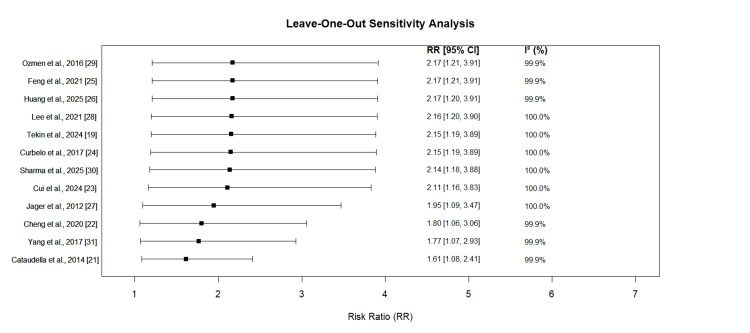
Sensitivity Analysis

Sensitivity analysis was conducted using the leave-one-out method, systematically removing each study individually and recalculating the pooled estimate and heterogeneity statistics (Figure 3). The analysis demonstrated that no single study significantly influenced the overall effect estimate or heterogeneity measures. Removal of any individual study did not substantially alter the pooled effect size or reduce the I² values, indicating that the observed heterogeneity was not driven by any single outlier but rather represents genuine variability across the included studies. This shows that heterogeneity is systemic across all studies.

Effect of NLR on ICU Admission

**Figure 4:**
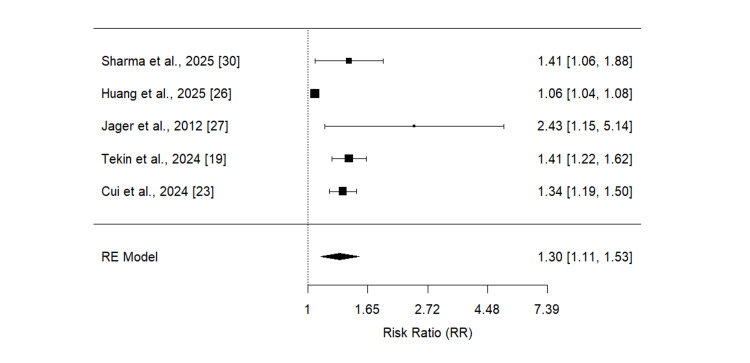
Effect of neutrophil-to-lymphocyte ratio (NLR) on ICU admission References [19, 23, 26-27, 30]

Five studies were included to evaluate the impact of NLR on ICU admission, with the pooled findings presented in Figure 4. The meta-analysis demonstrated that elevated NLR was significantly associated with an increased risk of ICU admission among patients with CAP (RR = 1.30, 95% CI: 1.11-1.53). However, substantial heterogeneity was observed across the included studies (I² = 85%).

Discussion

This meta-analysis of 12 studies involving patients with community-acquired pneumonia (CAP) demonstrates that elevated neutrophil-to-lymphocyte ratio (NLR) is significantly associated with increased all-cause mortality risk (RR: 2.02, 95% CI: 1.18 to 3.47). This finding suggests that NLR serves as a valuable prognostic biomarker in CAP management, with patients exhibiting high NLR values having approximately twice the risk of death compared to those with lower ratios. Meta-analysis conducted by Alzoubi and Khanfar reported significant difference in the NLR between survivors and non-survivor subjects [32].

Elevated NLR in CAP patients may indicate several adverse pathophysiological processes: (1) overwhelming systemic inflammatory response syndrome (SIRS), (2) relative immunosuppression with increased susceptibility to secondary infections, (3) endothelial dysfunction and microvascular compromise, and (4) increased risk of septic shock and multi-organ failure [33-34]. These mechanisms collectively contribute to the observed increased mortality risk. Neutrophils are responsible for the first line of host immune response against invading pathogens through chemotaxis, phagocytosis, and the release of cytokines, while also playing an important regulatory role in adaptive immunity [35].

Our findings are consistent with previous research demonstrating the prognostic value of NLR in various infectious and inflammatory conditions. The magnitude of risk (RR: 2.02) aligns with other studies showing NLR as a predictor of adverse outcomes in sepsis, where similar risk ratios have been reported [36-37]. A recent meta-analysis by Zhang et al. found that higher NLR was associated with poor prognosis in sepsis patients with a mean hazard ratio of 1.75 [38]. This consistency across different infectious conditions supports the biological plausibility of NLR as a universal marker of immune dysfunction and inflammation severity.

The prognostic performance of NLR in our analysis compares favorably with traditional severity scoring systems in pneumonia. While established tools like CURB-65 and PSI remain gold standards for CAP severity assessment [39], NLR offers advantages including widespread availability, low cost, and real-time assessment capability. Recent studies have demonstrated that adding NLR to conventional scoring systems significantly increases their discrimination for predicting mortality [25]. The integration of NLR with existing severity scores may enhance prognostic accuracy and guide clinical decision-making.

The substantial heterogeneity observed in our analysis (I² = 99%) reflects the complex nature of CAP as a clinical entity and the diverse methodological approaches across included studies. Sources of heterogeneity likely include variations in: (1) patient populations and comorbidity profiles, (2) pneumonia severity and etiology, (3) NLR cutoff values and measurement timing, (4) treatment protocols and healthcare settings, and (5) outcome definitions and follow-up periods [40].

The robust sensitivity analysis using the leave-one-out method confirmed that the observed heterogeneity was not driven by any single outlier study, supporting the validity of our pooled estimates. This heterogeneity, while challenging for interpretation, may actually reflect the real-world diversity of CAP patients and healthcare settings, potentially enhancing the generalizability of our findings. Similar heterogeneity has been reported in other meta-analyses examining inflammatory biomarkers in infectious diseases [41].

Several limitations must be acknowledged. The high heterogeneity limits the precision of pooled estimates and may reduce the applicability to specific patient subgroups. The observational nature of the included studies precludes causal inferences about the relationship between NLR and mortality. Variations in NLR measurement timing, cutoff values, and outcome definitions across studies may have influenced results. Furthermore, potential confounding by unmeasured variables, such as comorbidity severity or treatment variations, cannot be excluded. The lack of standardized NLR cutoff values across studies, with some using NLR >12 and others using different thresholds, may have contributed to heterogeneity.

Study Limitations

Several areas warrant further investigation to optimize the clinical utility of NLR in CAP management. Prospective studies are needed to establish standardized NLR cutoff values and optimal measurement timing. Research should focus on developing integrated prognostic models combining NLR with existing severity scores and other biomarkers. Recent studies have shown promise in combining NLR with platelet-to-lymphocyte ratio and other inflammatory markers for enhanced prognostic accuracy. The cost-effectiveness of NLR-guided management strategies requires a formal health economic evaluation. Additionally, studies examining NLR trends during treatment and their correlation with clinical response would provide valuable insights for dynamic risk stratification. Investigation of NLR performance in specific CAP subgroups, including immunocompromised patients and those with different pathogen etiologies, would enhance precision medicine approaches. Future research should also explore the role of low-density neutrophils and their contribution to NLR values in different disease states.

## Conclusions

This meta-analysis provides compelling evidence that an elevated neutrophil-to-lymphocyte ratio serves as a significant prognostic indicator for mortality in community-acquired pneumonia patients, with high NLR values associated with approximately double the death risk. The widespread availability, low cost, and real-time assessment capability of NLR make it an attractive biomarker for clinical practice. Despite substantial heterogeneity across studies reflecting diverse patient populations and methodological approaches, sensitivity analysis confirmed the robustness of our findings. Integration of NLR with existing severity scoring systems may enhance prognostic accuracy and guide clinical decision-making in CAP management. Future research should focus on establishing standardized NLR cutoff values, optimal measurement timing, and cost-effectiveness of NLR-guided treatment strategies to maximize its clinical utility in improving patient outcomes.

## References

[1] Torres A, Cilloniz C, Niederman MS, Menéndez R, Chalmers JD, Wunderink RG, van der Poll T (2021). Pneumonia. Nat Rev Dis Primers.

[2] Huang L, Weng B, Gu X (2024). Performance of various pneumonia severity models for predicting adverse outcomes in elderly inpatients with community-acquired pneumonia. Clin Microbiol Infect.

[3] Fine MJ, Auble TE, Yealy DM (1997). A prediction rule to identify low-risk patients with community-acquired pneumonia. N Engl J Med.

[4] Wedzicha W (2003). Airwaves. Thorax.

[5] Brito V, Niederman MS (2010). Predicting mortality in the elderly with community-acquired pneumonia: should we design a new car or set a new 'speed limit'?. Thorax.

[6] Parsonage M, Nathwani D, Davey P, Barlow G (2009). Evaluation of the performance of CURB-65 with increasing age. Clin Microbiol Infect.

[7] Schuetz P, Briel M, Bucher HC (2013). Procalcitonin to initiate or discontinue antibiotics in acute respiratory tract infections. Evid Based Child Health.

[8] Póvoa P, Martin-Loeches I, Ramirez P (2016). Biomarker kinetics in the prediction of VAP diagnosis: results from the BioVAP study. Ann Intensive Care.

[9] Zahorec R (2021). Neutrophil-to-lymphocyte ratio, past, present and future perspectives. Bratisl Lek Listy.

[10] Buonacera A, Stancanelli B, Colaci M, Malatino L (2022). Neutrophil to lymphocyte ratio: an emerging marker of the relationships between the immune system and diseases. Int J Mol Sci.

[11] Rosales C (2020). Neutrophils at the crossroads of innate and adaptive immunity. J Leukoc Biol.

[12] de Jager CP, van Wijk PT, Mathoera RB, de Jongh-Leuvenink J, van der Poll T, Wever PC (2010). Lymphocytopenia and neutrophil-lymphocyte count ratio predict bacteremia better than conventional infection markers in an emergency care unit. Crit Care.

[13] Lowsby R, Gomes C, Jarman I (2015). Neutrophil to lymphocyte count ratio as an early indicator of blood stream infection in the emergency department. Emerg Med J.

[14] Mantovani A, Cassatella MA, Costantini C, Jaillon S (2011). Neutrophils in the activation and regulation of innate and adaptive immunity. Nat Rev Immunol.

[15] Kuikel S, Pathak N, Poudel S, Thapa S, Bhattarai SL, Chaudhary G, Pandey KR (2022). Neutrophil-lymphocyte ratio as a predictor of adverse outcome in patients with community-acquired pneumonia: a systematic review. Health Sci Rep.

[16] Shan W, Shi T, Chen K (2019). Risk factors for severe community-aquired pneumonia among children hospitalized with CAP younger than 5 years of age. Pediatr Infect Dis J.

[17] Elassal GM, Elsayed MA, Shehata AM (2021). Value of neutrophil to lymphocyte ratio in prognosis of elderly patients with community acquired pneumonia compared to CRP level. QJM.

[18] Postma DF, van Werkhoven CH, van Elden LJ (2015). Antibiotic treatment strategies for community-acquired pneumonia in adults. N Engl J Med.

[19] Tekin A, Wireko FW, Gajic O, Odeyemi YE (2024). The neutrophil/lymphocyte ratio and outcomes in hospitalized patients with community-acquired pneumonia: a retrospective cohort study. Biomedicines.

[20] Wells G, Shea B, O’connell D, Peterson J, Welch V, Losos M, Tugwell P (2014). Newcastle-Ottawa quality assessment scale cohort studies. University of Ottawa.

[21] Cataudella E, Giraffa CM, Di Marca S (2017). Neutrophil-to-lymphocyte ratio: an emerging marker predicting prognosis in elderly adults with community-acquired pneumonia. J Am Geriatr Soc.

[22] Cheng S, Hou G, Liu Z (2020). Risk prediction of in-hospital mortality among patients with type 2 diabetes mellitus and concomitant community-acquired pneumonia. Ann Palliat Med.

[23] Cui XJ, Xie B, Zhu KW (2024). Prognostic value of the platelet, neutrophil, monocyte, basophil, and eosinophil to lymphocyte ratios in patients with severe community-acquired pneumonia (SCAP). Sci Rep.

[24] Curbelo J, Luquero Bueno S, Galván-Román JM (2017). Inflammation biomarkers in blood as mortality predictors in community-acquired pneumonia admitted patients: Importance of comparison with neutrophil count percentage or neutrophil-lymphocyte ratio. PLoS One.

[25] Feng DY, Zou XL, Zhou YQ, Wu WB, Yang HL, Zhang TT (2021). Combined neutrophil-to-lymphocyte ratio and CURB-65 score as an accurate predictor of mortality for community-acquired pneumonia in the elderly. Int J Gen Med.

[26] Huang L, Weng B, Wang M (2025). The improved prediction value of neutrophil to lymphocyte ratio to pneumonia severity scores for mortality in the older people with community-acquired pneumonia. BMC Geriatr.

[27] de Jager CP, Wever PC, Gemen EF, Kusters R, van Gageldonk-Lafeber AB, van der Poll T, Laheij RJ (2012). The neutrophil-lymphocyte count ratio in patients with community-acquired pneumonia. PLoS One.

[28] Lee H, Kim I, Kang BH, Um SJ (2021). Prognostic value of serial neutrophil-to-lymphocyte ratio measurements in hospitalized community-acquired pneumonia. PLoS One.

[29] Ozmen I, Karakurt Z, Salturk C (2016). Can N-terminal pro B-type natriuretic peptide, neutrophil-to-lymphocyte ratio, C-reactive protein help to predict short and long term mortality?. Bratisl Lek Listy.

[30] Sharma Y, Thompson C, Zinellu A, Shahi R, Horwood C, Mangoni AA (2025). The role of the neutrophil-to-lymphocyte ratio in predicting outcomes among patients with community-acquired pneumonia. Clin Med (Lond).

[31] Yang T, Wan C, Wang H, Qin J, Chen L, Shen Y, Wen F (2017). The prognostic and risk-stratified value of neutrophil-lymphocyte count ratio in Chinese patients with community-acquired pneumonia. Eur J Inflamm.

[32] Alzoubi O, Khanfar A (2022). Association between neutrophil to lymphocyte ratio and mortality among community acquired pneumonia patients: a meta-analysis. Monaldi Arch Chest Dis.

[33] File TM Jr, Ramirez JA (2023). Community-acquired pneumonia. N Engl J Med.

[34] Ye W, Chen X, Huang Y (2020). The association between neutrophil-to-lymphocyte count ratio and mortality in septic patients: a retrospective analysis of the MIMIC-III database. J Thorac Dis.

[35] Rosales C (2018). Neutrophil: a cell with many roles in inflammation or several cell types?. Front Physiol.

[36] Liu S, Wang X, She F, Zhang W, Liu H, Zhao X (2021). Effects of neutrophil-to-lymphocyte ratio combined with interleukin-6 in predicting 28-day mortality in patients with sepsis. Front Immunol.

[37] Qiu Y, Su Y, Tu GW (2020). Neutrophil-to-lymphocyte ratio predicts mortality in adult renal transplant recipients with severe community-acquired pneumonia. Pathogens.

[38] Wu H, Cao T, Ji T, Luo Y, Huang J, Ma K (2024). Predictive value of the neutrophil-to-lymphocyte ratio in the prognosis and risk of death for adult sepsis patients: a meta-analysis. Front Immunol.

[39] Lim WS, van der Eerden MM, Laing R (2003). Defining community acquired pneumonia severity on presentation to hospital: an international derivation and validation study. Thorax.

[40] Enersen CC, Egelund GB, Petersen PT (2023). The ratio of neutrophil-to-lymphocyte and platelet-to-lymphocyte and association with mortality in community-acquired pneumonia: a derivation-validation cohort study. Infection.

[41] Russell CD, Parajuli A, Gale HJ (2019). The utility of peripheral blood leucocyte ratios as biomarkers in infectious diseases: a systematic review and meta-analysis. J Infect.

